# Intravesical Therapy for Non-Muscle-Invasive Bladder Cancer: What Is the Real Impact of Squamous Cell Carcinoma Variant on Oncological Outcomes?

**DOI:** 10.3390/medicina58010090

**Published:** 2022-01-07

**Authors:** Guglielmo Mantica, Francesco Chierigo, Rafaela Malinaric, Salvatore Smelzo, Francesca Ambrosini, Martina Beverini, Giovanni Guano, Alberto Caviglia, Lorenzo Rigatti, Aldo Franco De Rose, Alessandro Tafuri, Davide De Marchi, Franco Gaboardi, Nazareno Suardi, Carlo Terrone

**Affiliations:** 1 Department of Urology, Policlinico San Martino Hospital, University of Genova, 16132 Genova, Italy; francesco.chierigo@gmail.com (F.C.); rafaela.malinarc@gmail.com (R.M.); f.ambrosini1@gmail.com (F.A.); martina.beverini@live.it (M.B.); giovanni.guano@gmail.com (G.G.); caviglialberto@gmail.com (A.C.); aldofrancoderose@gmail.com (A.F.D.R.); suardi.nazareno@gmail.com (N.S.); carlo.terrone@med.uniupo.it (C.T.); 2 Department of Urology, San Raffaele Turro Hospital, 20127 Milan, Italy; salvatore.smelzo@hsr.it (S.S.); rigatti.lorenzo@hsr.it (L.R.); demarchidavide82@gmail.com (D.D.M.); gaboardi.franco@hsr.it (F.G.); 3 Department of Urology, Vito Fazzi Hospital, 73110 Lecce, Italy; aletaf@hotmail.it

**Keywords:** squamous cell carcinoma, urinary bladder neoplasms, regression analysis, neoplasm recurrence, adjuvants

## Abstract

*Background and Objectives*: To evaluate the oncological impact of squamous cell carcinoma (SCC) variant in patients submitted to intravesical therapy for non-muscle-invasive bladder cancer (NMIBC). *Materials and Methods*: Between January 2015 and January 2020, patients with conventional urothelial NMIBC (TCC) or urothelial NMIBC with SCC variant (TCC + SCC) and submitted to adjuvant intravesical therapies were collected. Kaplan–Meier analyses targeted disease recurrence and progression. Uni- and multivariable Cox regression analyses were used to test the role of SCC on disease recurrence and/or progression. *Results*: A total of 32 patients out of 353 had SCC at diagnosis. Recurrence was observed in 42% of TCC and 44% of TCC + SCC patients (*p* = 0.88), while progression was observed in 12% of both TCC and TCC + SCC patients (*p* = 0.78). At multivariable Cox regression analyses, the presence of SCC variant was not associated with higher rates of neither recurrence (*p* = 0.663) nor progression (*p* = 0.582). *Conclusions*: We presented data from the largest series on patients with TCC and concomitant SCC histological variant managed with intravesical therapy (BCG or MMC). No significant differences were found in term of recurrence and progression between TCC and TCC + SCC. Despite the limited sample size, this study paves the way for a possible implementation of the use of intravesical BCG and MMC in NMIBC with histological variants.

## 1. Introduction

Bladder cancer (BCa) is one of the most common malignancies, and it counts for about 165,000 deaths/year worldwide [1,2,3]. Overall, 75% of newly diagnosed BCas are non-muscle-invasive (NMIBC), which is a disease burdened by recurrence in more than 60% of cases and progression in more than 10% [4,5]. The most predominant histological phenotype is the conventional urothelial carcinoma/Transitional Cell Carcinoma (TCC), which constitutes about 80% of bladder cancer. Histological variants (HVs) [6,7] are present in up to 20% of patients, and the squamous cell carcinoma (SCC) variant in roughly 10% of patients [8]. The role of HVs in NMIBC has become of great interest over the last decade. The presence of HVs is generally associated with a worse prognosis and a high risk of under-staging at the transurethral resection of the bladder tumor (TURBT) because of the small sample sizes and tumor heterogeneity. Urothelial variants present with histological features in common with conventional urothelial carcinoma and also display foci of specific different histomorphological phenotypes. Highly aggressive HVs are sarcomatoid carcinoma, plasmacytoid carcinoma, and micropapillary carcinoma. Furthermore, squamous and glandular differentiation show aggressive behavior. Nested, small tubular, microcystic, and inverted papilloma-like variants have been grouped under the heading of deceptively benign carcinomas because of their bland appearance and low-grade feature. Early radical cystectomy (RC) is often proposed in the majority of cases of high-grade (HG) NMIBC, although whether or not early radical cystectomy should be recommended has not been established yet. Squamous or glandular differentiation, deceptively benign carcinomas, and lymphoepithelioma such as carcinoma can initially be managed with restaging TURB followed by intravesical BCG (Bacillus Calmette-Guérin). However, any other urothelial or nonurothelial variants should be promptly considered for early radical cystectomy [4,9,10,11].

However, there is still little evidence on the impact of these variants on prognosis, recurrence, and management of BCa [9].

Currently, literature is scant of studies evaluating the efficacy and outcomes of adjuvant intravesical therapy in patients with HV [9].

Most of the very few published reports are focused on glandular or squamous variants, based on a series of 6–30 cases, and comparing patients managed with intravesical Bacillus Calmette–Guerin (BCG) to those submitted to RC [11,12,13,14,15].

These studies showed that both intravesical BCG and RC lead to a survival gain in high-risk patients diagnosed with NMIBC with squamous or glandular histologic variants and that RC was not mandatory for all of them [12,13].

A recent study by Gofrit et al. [16] compared the oncological outcomes of 41 patients with high-grade (HG) NMIBC + HV (any type) managed with intravesical BCG to those with conventional HG NMIBC. A total of 14 patients were diagnosed with micropapillary differentiation, 13 patients with squamous differentiation, 9 patients with glandular differentiation, and 7 patients with nested variants. The 2- and 5-year survival rates of the four variants were quite similar to each other. Patients with the glandular variant had a tendency toward a better prognosis, while patients with the micropapillary and squamous variants exhibited a worse prognosis. As compared with conventional urothelial carcinoma, patients with HV have a significantly worse prognosis, including 5-year, recurrence-free survival; 5-year progression-free survival; and overall survival. However, the series accounted for only 13 patients with squamous differentiation [16]. 

Within this paucity of data, the aim of this study is to analyze the efficacy of adjuvant intravesical chemotherapy (BCG or Mitomycin C (MMC)) in patients with NMIBC and SCC variant in a larger series. 

## 2. Materials and Methods

### 2.1. Study Design and Patient Population

Between January 2015 and January 2020, data of all patients affected by NMIBC and submitted to adjuvant intravesical therapies in two tertiary care referral centers were prospectively collected in our multi-institutional databases and retrospectively analyzed. Anamnestic information such as age and gender and clinicopathological data (histology at TURBT, American Joint Committee on Cancer (TNM) staging) available at the time of the adjuvant intravesical therapy were recorded. We included patients with TURBT pathological reports showing a conventional urothelial NMIBC (TCC) or urothelial NMIBC with any percentage of associated SCC variant (TCC + SCC), alone or in combination with Carcinoma in situ (CIS), with intermediate or high-risk of recurrence and progression [4]. 

No blue-light cystoscopy and narrow-band imaging as diagnostic tools were used. 

All patients were treated with adjuvant intravesical therapy (either BCG or MMC) according to the current European Association of Urology (EAU) guidelines [17]. Only patients submitted to TURBT at our institutions were included in the study. Patients who underwent a single early postoperative instillation of chemotherapy or those who did not complete the induction were excluded. Patients with incomplete information were excluded from the study.

All the TURBT specimens were analyzed by uropathologists of the same pathology department. Diagnosis of urothelial bladder carcinoma with rare SCC variant differentiation was based on the identification of specific histological features according to the World Health Organization classification in use at the time of diagnosis. SCC can be described as an epithelial neoplasm exclusively displaying histological features such as squamous pearls, intercellular bridges, or keratinization [18]. Molecular squamous markers used were CK5/6, p63, p40, CK14, and desmoglein 3. Pathological staging was established according to the TNM system [19].

### 2.2. Adjuvant Intravesical Therapy and Follow-Up

The adjuvant intravesical MMC was administered according to the Southwest Oncology Group (SWOG) regimen: induction with a weekly injection per 6 weeks + maintenance with a monthly injection up to 12 months. Similarly, the BCG scheme included a weekly injection up to 6 weeks and a maintenance every 3–6 months up to 36 months [17]. 

Patients were followed-up with cystoscopy, ultrasound, and urine cytology according to EAU guidelines recommendations [4]. The exact date of disease recurrence and/or progression was recorded. We evaluated the differences in terms of age, stage, grade, and risk factors and compared such variables between TCC and TCC + SCC patients. Moreover, the response to intravesical therapy (BCG or MMC) was evaluated in both TCC and TCC + SCC patients in terms of disease recurrence and/or progression. Disease progression was defined as any upgrade to a higher pathological T (pT) stage. We considered the follow-up time starting from the histological diagnosis. 

### 2.3. Statistical Analysis

Data were entered into a Microsoft Excel (version 14.0) (Microsoft Corporation, Redmond, WA, USA)database and transferred to SPSS v.24.0 for Windows (IBM Corp., Armonk, NY, USA). Continuous and non-normally distributed variables are presented as medians with interquartile ranges (IQRs). For the comparison between demographic and pathological data Mann–Whitney and Chi-squared tests were used for continuous and categorical variables, respectively. Kaplan–Meier analyses targeted disease recurrence and progression according to the presence of SCC as well as according to the different intravesical drugs. Finally, uni- and multivariable Cox regression analyses were used to test the role of SCC on disease recurrence and/or progression.

## 3. Results

Overall, a total of 353 patients were evaluated. Of these, 32 (9.1%) had SCC at diagnosis. No significant differences in terms of mean age, smoking, pT1 stage, high grade, concomitant carcinoma in situ, dimensions, and single vs. multiple lesions were found between TCC + SCC and TCC patients, respectively (Table 1). Three patients (0.9%) were pT2 at the TURBT and underwent intravesical therapy, having refused RC. Intravesical therapy with MMC was administered in 15 (47%) TCC + SCC vs. 163 (51%) TCC patients, respectively. Intravesical therapy with BCG was administered in 17 (53%) TCC + SCC vs. 142 (44%) TCC patients, respectively. Furthermore, 16 TCC patients underwent epirubicin (Table 1).

**Figure 1:**
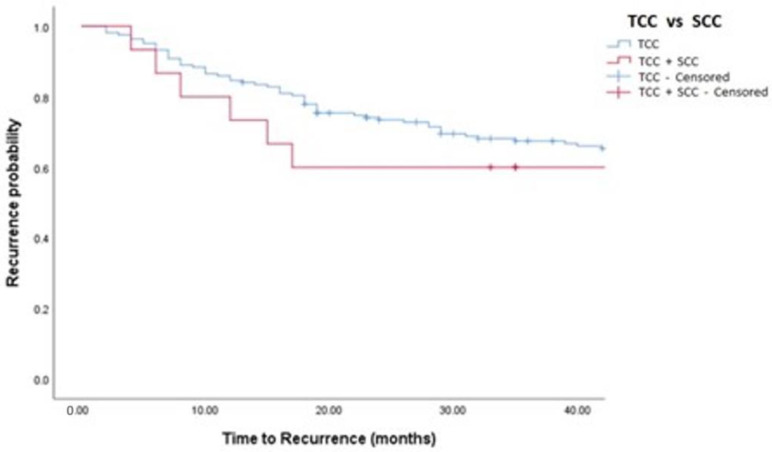
Kaplan–Meier analysis showing estimates of disease recurrence in TCC and TCC + SCC patients treated with MMC (log-rank *p* = 0.448). TCC = Conventional Urothelial non-muscle-invasive bladder cancer. SCC = Squamous Cell Carcinoma.

**Figure 2:**
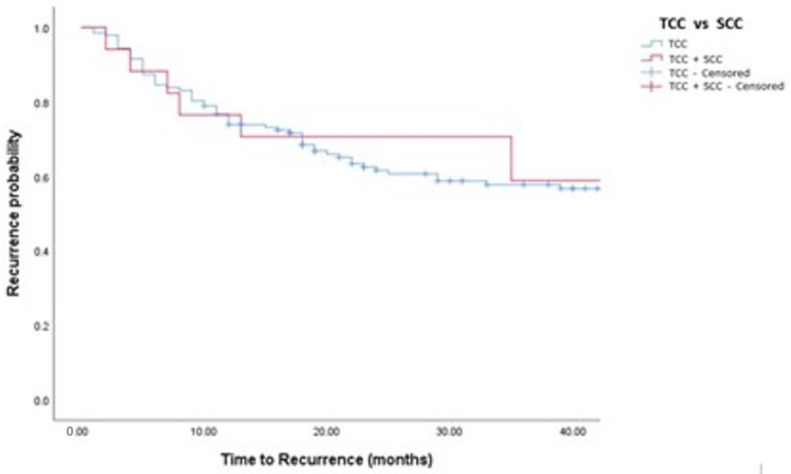
Kaplan–Meier analysis showing estimates of disease recurrence in in TCC and TCC + SCC patients treated with BCG (log-rank *p* = 0.532).

**Figure 3:**
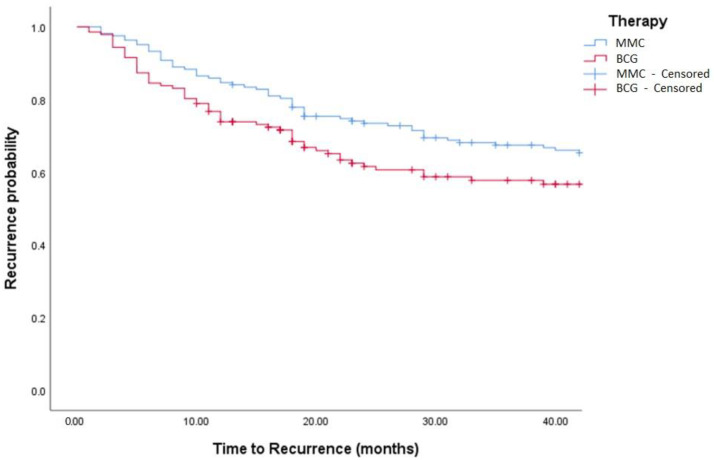
Kaplan–Meier in BCG vs. MMC in TCC patients (log-rank, *p* = 0.271). BCG = Bacillus Calmette Guerin. MCC = Mitomycin C.

**Figure 4:**
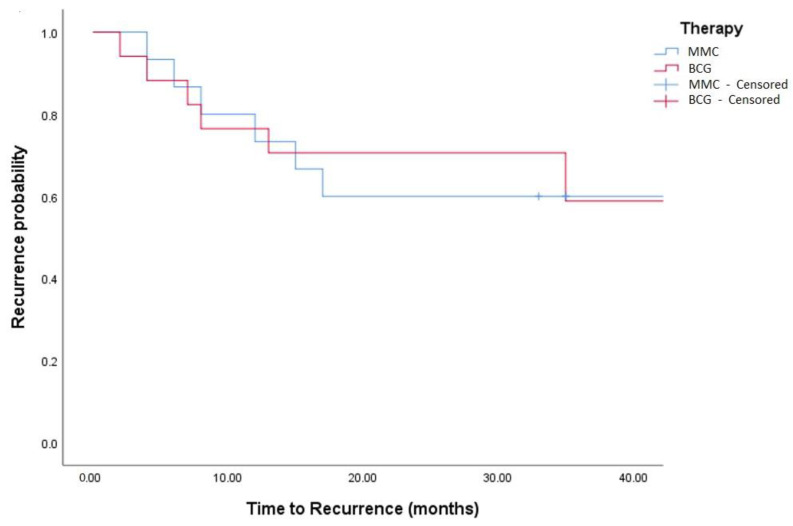
Kaplan–Meier in TCC + SCC patients (log-rank, *p* = 0.759).

Median follow-up was 36 (IQR: 18–50) months. Oncological outcomes are shown in Table 1, Table 2 and Table 3 and Figure 1, Figure 2, Figure 3 and Figure 4. Recurrence was observed in 42% of TCC and 44% of TCC + SCC patients (*p* = 0.88), while progression was observed in 12% of both TCC and TCC + SCC patients (*p* = 0.78). Patients treated with MMC showed a 1- and 3- year recurrence-free survival (RFS) of 74% and 51% for TCC and 60% and 43% in TCC + SCC, respectively. Patients treated with BCG showed a 1- and 3- year RFS of 62% and 42% for TCC and 71% and 59% for TCC + SCC, respectively.

At multivariable Cox regression analyses, after adjusting for age, pT stage, and grade at diagnosis as well as the type of intravesical therapy, the presence of SCC variant at TURBT was associated with higher rates of neither recurrence (*p* = 0.663) nor progression (*p* = 0.582) (Table 2 and Table 3). 

## 4. Discussion

BCa HV, and therefore also SCC, are usually considered to have a poor prognosis, and patients are generally submitted to aggressive treatments, such as early cystectomy [9,20]. Currently, literature is scant of studies evaluating the efficacy and outcomes of adjuvant intravesical therapy in patients with HV. Most of these few studies, mainly based on less than a dozen of patients with glandular or squamous variants, showed that both intravesical BCG and RC lead to a survival gain in high-risk patients diagnosed with NMIBC bladder cancer with squamous or glandular histologic variants and that RC is not mandatory for every patient [12,13].

Our study represents, currently, the largest series on patients with SCC variant managed with intravesical therapy. At a median follow-up of 36 months, no significant differences were found in terms of recurrence and progression between TCC and TCC + SCC patients. 

Patients treated with MMC showed a 1- and 3- year RFS of 74% and 51% for TCC and 60% and 43% in TCC + SCC, respectively. Patients treated with BCG showed a 1- and 3- year RFS of 62% and 42% for TCC and 71% and 59% for TCC + SCC, respectively.

At multivariable Cox regression analyses, after adjusting for age, pT stage, and grade at diagnosis as well as the type of intravesical therapy, the presence of SCC variant at TUR was associated with higher rates of neither recurrence (*p* = 0.663) nor progression (*p* = 0.582).

Anecdotally, looking at other fields, in particular regarding squamous cell carcinoma, irrigation and topical use of MMC and BCG are successfully reported in selected cases of squamous cell carcinoma of the head and neck or lung [21,22,23]. Similarly, BCG is widely used in the veterinary field for the management of ocular squamous cell carcinoma in bovines [24,25].

This study has several limitations. Firstly, the possible biases of a multicentric study, with different operators administering the intravesical therapy and following the patients. Secondly, although the data were collected prospectively, there was no randomization of patients. Furthermore, the control group over the same time period appears much larger than the study group. Third, there may have been a selection bias of patients undergoing intravesical instillation rather than early cystectomy. Fourth, the percentage of SCC variant on histology is heterogeneous, and it could impact the results. However, given the limited sample size and the short follow-up, it is impossible to statistically evaluate the impact with these data. 

Almost no data are available in literature on intravesical management of TCC with HV, mainly due to the aggressiveness of the neoplasm for which urologists tend to recommend more aggressive management such as early cystectomy. Despite the numerous concerns, these preliminary data could lead at least to considering intravesical therapy in patients with TCC associated with some variants, in particular SCC.

## 5. Conclusions

We presented data from the largest series on patients with TCC and concomitant SCC histological variant managed with intravesical therapy (BCG or MMC). At a median follow-up of 36 months, no significant differences were found in terms of recurrence and progression between TCC and TCC + SCC. Furthermore, the presence of the SCC variant at TURBT was associated with neither higher recurrence nor progression rates. Despite the limited sample size and numerous biases, this study paves the way for a possible implementation of the use of intravesical BCG and MMC in NMIBC with HV. Further, larger multicentric and randomized trials are needed to confirm these preliminary data.

## Figures and Tables

**Table 1:** Clinical and pathological characteristics at diagnosis and recurrence.

Characteristic	Overall, *n* = 353	TCC, *n* = 321	TCC + SCC, *n* = 32	*p*-Value
Age	72 (65, 78)	72 (65, 78)	72 (66, 79)	0.72
Smoking, *n* (%)				0.10
No	42 (12%)	40 (12%)	2 (6.2%)	
Yes	196 (56%)	182 (57%)	14 (44%)	
Ex	115 (33%)	99 (31%)	16 (50%)	
Single vs. Multiple, *n* (%)				0.60
Single	203 (58%)	186 (58%)	17 (53%)	
Multiple	150 (42%)	135 (42%)	15 (47%)	
Dimensions, *n* (%)				0.40
<3 cm	196 (56%)	176 (55%)	20 (62%)	
≥3 cm	157 (44%)	145 (45%)	12 (38%)	
Histology, *n* (%)				0.57
pTa	186 (53%)	172 (54%)	14 (44%)	
pT1	144 (41%)	127 (40%)	17 (53%)	
pT2	3 (0.8%)	3 (0.9%)	0 (0%)	
Cis	19 (5.4%)	18 (5.6%)	1 (3.1%)	
Tx	1 (0.3%)	1 (0.3%)	0 (0%)	
Grading, *n* (%)				0.40
Low	100 (28%)	93 (29%)	7 (22%)	
High	253 (72%)	228 (71%)	25 (78%)	
Concomitant Cis	10 (2.8%)	9 (2.8%)	1 (3.1%)	>0.99
Therapy, *n* (%)				0.65
MMC	178 (50%)	163 (51%)	15 (47%)	
BCG	159 (45%)	142 (44%)	17 (53%)	
Epirubicin	16 (4.5%)	16 (4.9%)	0 (0%)	
Histology at Recurrence, *n* (%)				0.066
Ta	98 (64%)	91 (65%)	7 (50%)	
T1	33 (22%)	29 (21%)	4 (29%)	
T2	4 (2.6%)	2 (1.4%)	2 (14%)	
Cis	18 (12%)	17 (12%)	1 (7.1%)	
Tx	0 (0%)	0 (0%)	0 (0%)	
Grading at Recurrence, *n* (%)				0.29
Low	64 (42%)	60 (43%)	4 (29%)	
High	89 (58%)	79 (57%)	10 (71%)	
Concomitant Cis at Recurrence	5 (3.3%)	4 (2.9%)	1 (7.1%)	0.39
Time To Recurrence	35 (15, 58)	33 (16, 57)	40 (13, 78)	0.44
Recurrence, *n* (%)	150 (42%)	136 (42%)	14 (44%)	0.88
Progression, *n* (%)	42 (12%)	38 (12%)	4 (12%)	0.78
Time To Progression	36 (16, 58)	36 (16, 57)	40 (13, 78)	0.51

CI = confidence interval. TCC = Transitional Cell Carcinoma. SCC = squamous cell carcinoma. Cis = carcinoma in situ. BCG = Bacillus Calmette–Guerin. MMC = Mitomycin C.

**Table 2:** Multivariable Cox regression analysis predicting recurrence.

	Univariable		Multivariable	
	HR (95% CI)	*p*-Value	HR (95% CI)	*p*-Value
Age	1.00 (0.99, 1.01)	0.896	1.00 (0.99, 1.01)	0.947
Smoking (No as Ref)		0.592		0.643
Yes	0.96 (0.58, 1.59)	0.872	0.93 (0.55, 1.58)	0.792
Ex-smoker	1.16 (0.68, 1.97)	0.591	1.11 (0.63, 1.96)	0.710
Single vs. Multi (Single as Ref)	1.07 (0.76, 1.49)	0.702	1.07 (0.76, 1.53)	0.691
Dimension (<3 cm as Ref)	0.99 (0.71, 1.38)	0.958	1.01 (0.71, 1.44)	0.948
TCC vs. TCC + SCC	1.03 (0.59, 1.79)	0.915	0.88 (0.50, 1.56)	0.663
Histology (Ta as Ref)		0.041		0.033
T1	1.59 (0.64, 3.95)	0.315	1.30 (0.85, 2.00)	0.229
T2	1.76 (0.71, 4.41)	0.224	5.20 (1.53, 17.69)	0.008
Cis	7.48 (1.77, 31.60)	0.006	0.73 (0.28, 1.92)	0.528
Grading (Low as Ref)	0.79 (0.55, 1.12)	0.183	0.52 (0.31, 0.88)	0.015
Concomitant Cis (No as Ref)	1.22 (0.52, 2.87)	0.653	1.20 (0.51, 2.87)	0.674
BCG vs. MMC (MMC as Ref)	1.17 (0.84, 1.64)	0.362	1.47 (0.93, 2.35)	0.103

CI = confidence interval. TCC = Transitional Cell Carcinoma. SCC = squamous cell carcinoma. Cis = carcinoma in situ. BCG = Bacillus Calmette–Guerin. MMC = Mitomycin C.

**Table 3:** Multivariable Cox regression analysis predicting progression.

	Univariable		Multivariable	
	HR (95% CI)	*p*-Value	HR (95% CI)	*p*-Value
Age	1.01 (0.99, 1.04)	0.369	1.01 (0.99, 1.04)	0.303
Smoking (No as Ref)		0.118		0.090
Yes	1.21 (0.41, 3.58)	0.728	1.20 (0.39, 3.64)	0.753
Ex-smoker	2.26 (0.75, 6.83)	0.147	2.40 (0.76, 7.58)	0.135
Single vs. Multi (Single as Ref)	0.94 (0.50, 1.79)	0.856	0.80 (0.40, 1.57)	0.509
Dimension (<3 cm as Ref)	1.06 (0.57, 1.95)	0.865	1.03 (0.54, 1.97)	0.936
TCC vs. TCC + SCC	1.06 (0.38, 3.01)	0.907	0.74 (0.25, 2.17)	0.582
Histology (Ta as Ref)		0.072		0.298
T1	1.88 (0.97, 3.65)	0.061	1.67 (0.76, 3.68)	0.203
T2	9.42 (1.21, 73.23)	0.032	6.48 (0.75, 55.63)	0.089
Cis	1.89 (0.55, 6.55)	0.316	1.82 (0.46, 7.10)	0.392
Grading (Low as Ref)	0.55 (0.24, 1.26)	0.158	0.88 (0.29, 2.66)	0.814
Concomitant Cis (No as Ref)	1.78 (0.23, 13.90)	0.58	0.41 (0.05, 3.21)	0.397
BCG vs. MMC (MMC as Ref)	2.08 (1.09, 3.97)	0.026	1.81 (0.79, 4.15)	0.159

CI = confidence interval. TCC = Transitional Cell Carcinoma. SCC = squamous cell carcinoma. Cis = carcinoma in situ. BCG = Bacillus Calmette–Guerin. MMC = Mitomycin C.

## Data Availability

Data are available at the Institutional Database.
